# Prognosis of air quality index and air pollution using machine learning techniques

**DOI:** 10.1038/s41598-025-11260-y

**Published:** 2025-07-17

**Authors:** Mostafa M. Abdelmalek, Hatem Mahmoud, Hassan Shokry

**Affiliations:** 1 Environmental Engineering Department, Egypt-Japan University of Science and Technology, Alexandria, 21934 Egypt; 2 Mining and Metallurgical Engineering Department, Faculty of Engineering, Assiut University, Assiut, 71516 Egypt; 3 Department of Architecture Engineering, Engineering Faculty, Aswan University, Aswan, 81542 Egypt

**Keywords:** Environmental chemistry, Environmental impact

## Abstract

Air pollution constitutes a significant challenge for both public health and environmental sustainability. Pollutants like PM, O_3_, NO_2_, SO_2_, and CO cause serious health problems and ecological damage. This study utilizes five machine learning (ML) models, which are Gaussian Process Regression (GPR), Ensemble Regression (ER), Support Vector Machine (SVM), Regression Tree (RT), and Kernel Approximation Regression (KAR), which are developed and compared to predict the Air Quality Index (AQI). The publicly available historical air pollution dataset, collected from 1st January to 31st December 2022, was obtained from the online source titled ‘A Real-time Dataset of Air Pollution Monitoring Generated Using IoT—Mendeley Data’, developed by the Department of Software Engineering, Daffodil International University. While the dataset includes six pollutants (PM_10_, PM_2.5_, NO_2_, SO_2_, CO, and O_3_), only three—PM_2.5_, PM_10_, and CO—were selected for AQI prediction based on their higher feature importance as determined using the Random Forest technique. To streamline the time and cost consumed in measuring and analyzing these pollutants, the five ML models were employed to predict the AQI using only these three essential features. The findings reveal that GPR, ER, SVM, and RT ML models exhibited higher accuracy levels, achieving over 96% AQI prediction, whereas the KAR model was less accurate, with an accuracy of 82.36%. The comparative analysis revealed that the GPR model outperformed the other ML models with a minimum Root Mean Square Error (RMSE) of 0.87 and 1.219 during the training and testing, respectively. The findings highlight the value of ML in enhancing air quality prediction and monitoring, offering accurate tools for hourly data analysis and potential real-time application. Such tools can assist in devising more efficient air pollution control strategies, contributing to improved public health and environmental sustainability.

## Introduction

Air pollution is one of the most significant threats to public health globally, contributing to over seven million premature deaths each year, including 4.2 million due to outdoor air pollutants and 3.8 million from indoor exposure^1–3^. Developing countries such as Egypt and Bangladesh face particularly acute challenges driven by rapid urbanization, industrial growth, traffic congestion, and limited air quality monitoring infrastructure^4–7^. In Cairo, ambient air pollution was linked to approximately 19,200 premature deaths in 2017 alone^6^.

Air pollutants, including particulate matter (PM_2.5_ and PM_10_), carbon monoxide (CO), sulfur dioxide (SO_2_), nitrogen oxides (NO_x_), and ozone (O_3_), contribute to environmental and health hazards^4,8–10^. The Air Quality Index (AQI), derived from the concentration of these pollutants, provides a standardized framework to communicate air quality status and associated health risks^11–13^. Despite the AQI’s importance, traditional models rely on extensive pollutant and meteorological data, often increasing cost, complexity, and resource requirements.

Many studies have adopted ML models for AQI prediction across various urban regions, leveraging multiple pollutants and meteorological parameters^14–18^. For instance, RF, SVM, and Ensemble models have been used to predict AQI in cities such as Makkah^19^, California^20^, and Beijing^21^. Showing high accuracy but depends on six or more input features. While these models are effective, their real-world applicability in resource-constrained environments is limited.

Predicting air quality efficiently is critical for public health planning, particularly in developing countries with limited monitoring resources. Traditional AQI forecasting models often rely on a wide range of pollutant and meteorological inputs, increasing complexity and cost. This study proposes a simplified machine learning approach to predict AQI using only three main pollutants—PM_2.5_, PM_10_, and CO—derived from real-world data in Bangladesh. This choice reflects a balance between prediction accuracy and practical feasibility. While the authors are based in Egypt, the insights gained from this work using the Bangladeshi dataset aim to support scalable air quality assessment methods applicable across similar contexts in developing nations.

The main objective of this study is to develop a machine learning-based framework for accurate and cost-effective AQI prediction by identifying and utilizing the most influential air pollutants. A secondary objective is to compare the predictive performance of five advanced machine learning algorithms—GPR, ER, SVM, RT, and KAR—based on their effectiveness in estimating AQI. The study aims to provide a scalable and efficient model for real-world air quality monitoring applications, particularly in resource-constrained environments.

## Materials and methods

**Fig. 1:**
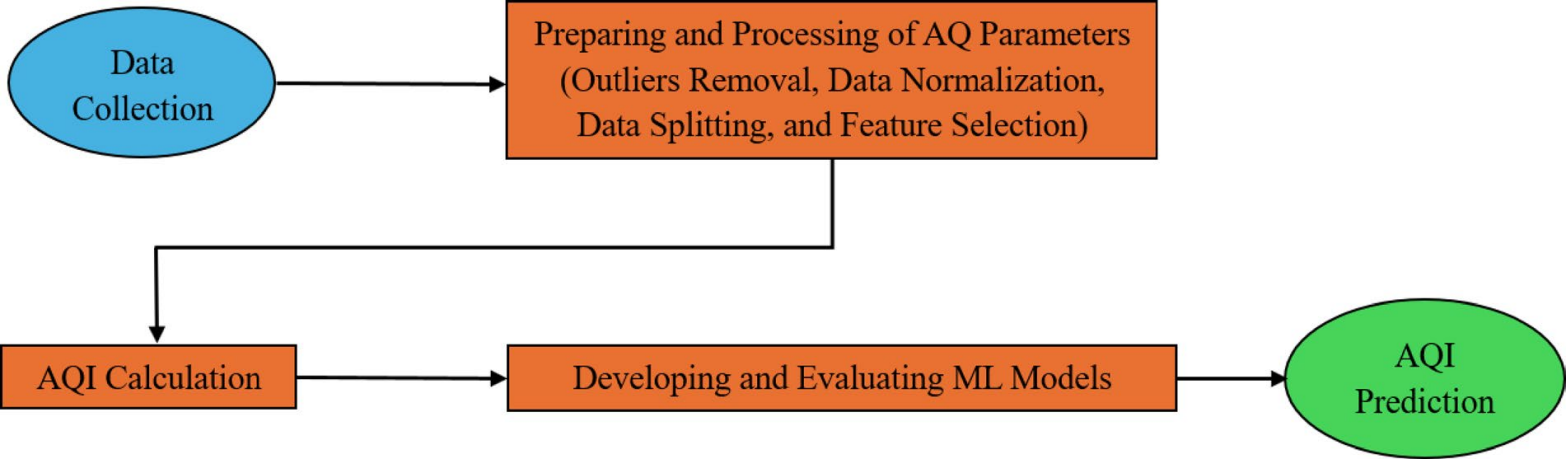
A flowchart illustrating the machine learning approach for AQI prediction.

This research has progressed through three main stages. The first one included preparing and processing air quality parameters. The second stage comprises calculating the AQI. Finally, developing and evaluating ML models. The adopted framework has been presented in Fig. 1.

### Data preparation and processing

The air pollution data utilized in this study is accessible online at A Real-time Dataset of Air Pollution Monitoring Generated Using IoT—Mendeley Data^22^. This dataset was collected hourly from 1st January 2022 to 31st December 2022 in Gazipur, Bangladesh, using an IoT-based monitoring system. It includes concentration levels of six pollutants: PM_2.5_, PM_10_, CO, NO_2_, SO_2_, and O_3_, which were used to compute the Air Quality Index (AQI). The AQI was calculated following the methodology of the U.S. Environmental Protection Agency (EPA), using the linear interpolation formula and the national air quality breakpoints adopted by the Department of Environment (DoE) in Bangladesh (see Table 1). For each pollutant, the sub-index ${I}_{p}$ was calculated using Eq. (1)^23^, and the overall AQI was determined as the maximum computed sub-indices for the six pollutants.

1 $${I}_{p}= \frac{{I}_{high}-{I}_{low}}{{C}_{high}- {C}_{low}}\left({C}_{p}- {C}_{low}\right)+ {I}_{low}$$

where:

**Table 1:** AQI Standards by DoE, Bangladesh.

AQI Category	PM_10_	PM_2.5_	NO_2_	O_3_	CO	SO_2_
	24 h	24 h	24 h	24 h	8 h	24 h
Good (0–50)	0–50	0–30	0–40	0–50	0–1	0–40
Satisfactory (51–100)	51–100	31–60	41–80	51–100	1.1–2.0	41–80
Moderate (101–200)	101–250	61–90	81–180	101–168	2.1–10	81–380
Poor (201–300)	251–350	91–120	181–280	169–208	10.1–17	381–800
Very Poor (301–400)	351–430	121–250	281–400	209–748	17.1–34	801–1600
Severe (401–500)	430 +	250 +	400 +	748 +	34 +	1600 +

${I}_{p}$= The AQI value corresponding to the pollutant *p.*

${C}_{p}$= The measured concentration of pollutant* p*

${C}_{low}$ = The threshold of the concentration that is ≤ ${C}_{p}$

${C}_{high}$ = The threshold of the concentration that is ≥ ${C}_{p}$

${I}_{low}$ = The index threshold associated with ${C}_{low}$

${I}_{high}$ = The index threshold associated with ${C}_{high}$

**Fig. 2:**
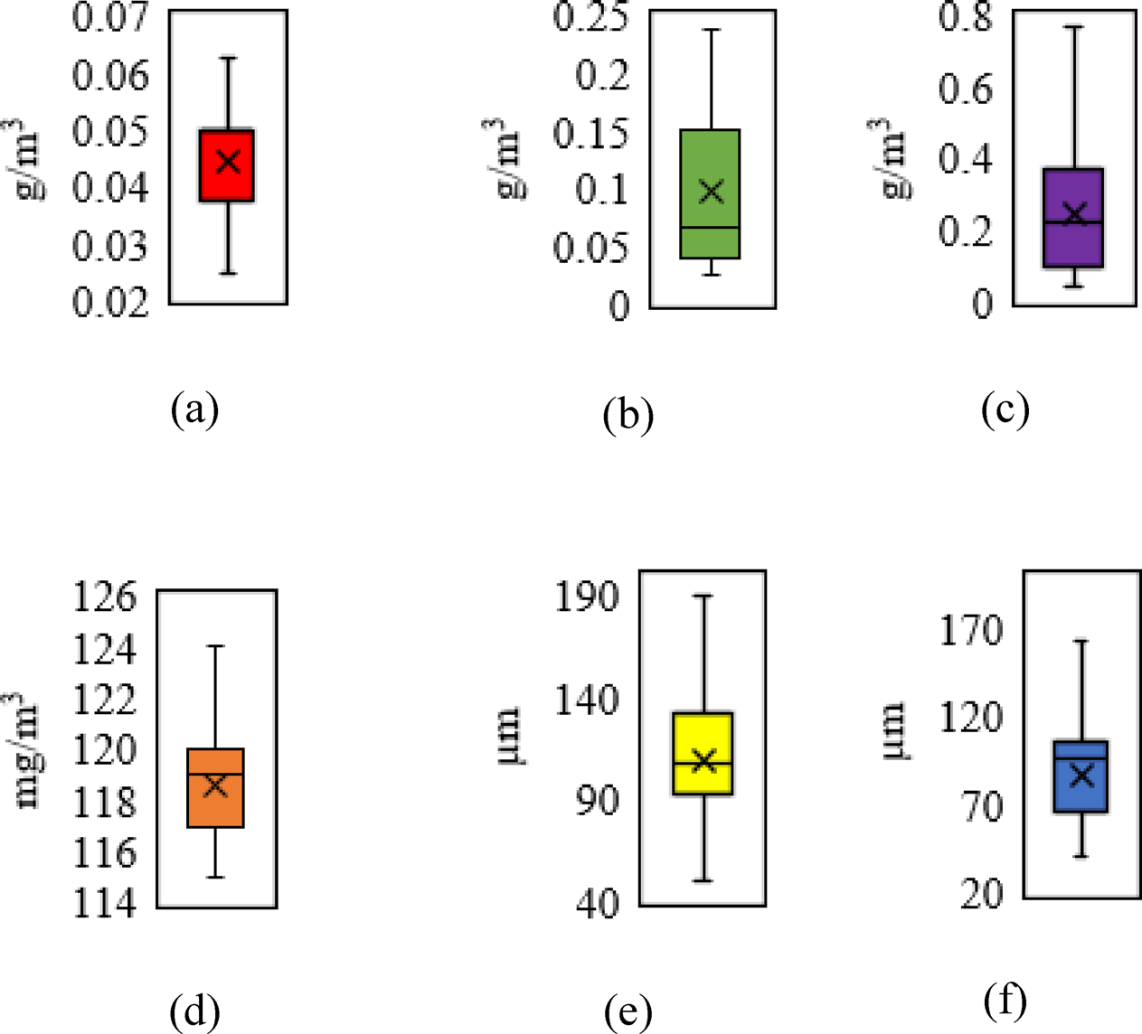
Box plotting of input and output parameters: (**a**) sulfur dioxide, (**b**) nitrogen dioxide, (**c**) ozone, (**d**) carbon monoxide, (**e**) particulate matter (D ≤ 2.5 µm), and (f) particulate matter (2.5 µm ≤ D ≤ 10 µm).

To ensure data quality, box plotting (Fig. 2) was first applied to identify and remove outliers from the raw concentration values of each pollutant. Each box plot displays the distribution of one pollutant using its actual measurement unit: PM_2.5_ and PM_10_ (μm), CO (mg/m^3^), and SO_2_, NO_2_, and O_3_ (g/m^3^). Following outlier removal, all variables were normalized to a range between 0 and 1 using the min–max scaling technique, which preserved the original distribution shapes while bringing the features into a comparable scale suitable for machine learning algorithms. The cleaned dataset was split into 80% for training and 20% for testing. To reduce sampling bias and improve generalizability, training, and testing were repeated multiple times, and tenfold cross-validation was conducted to evaluate model stability.

**Fig. 3:**
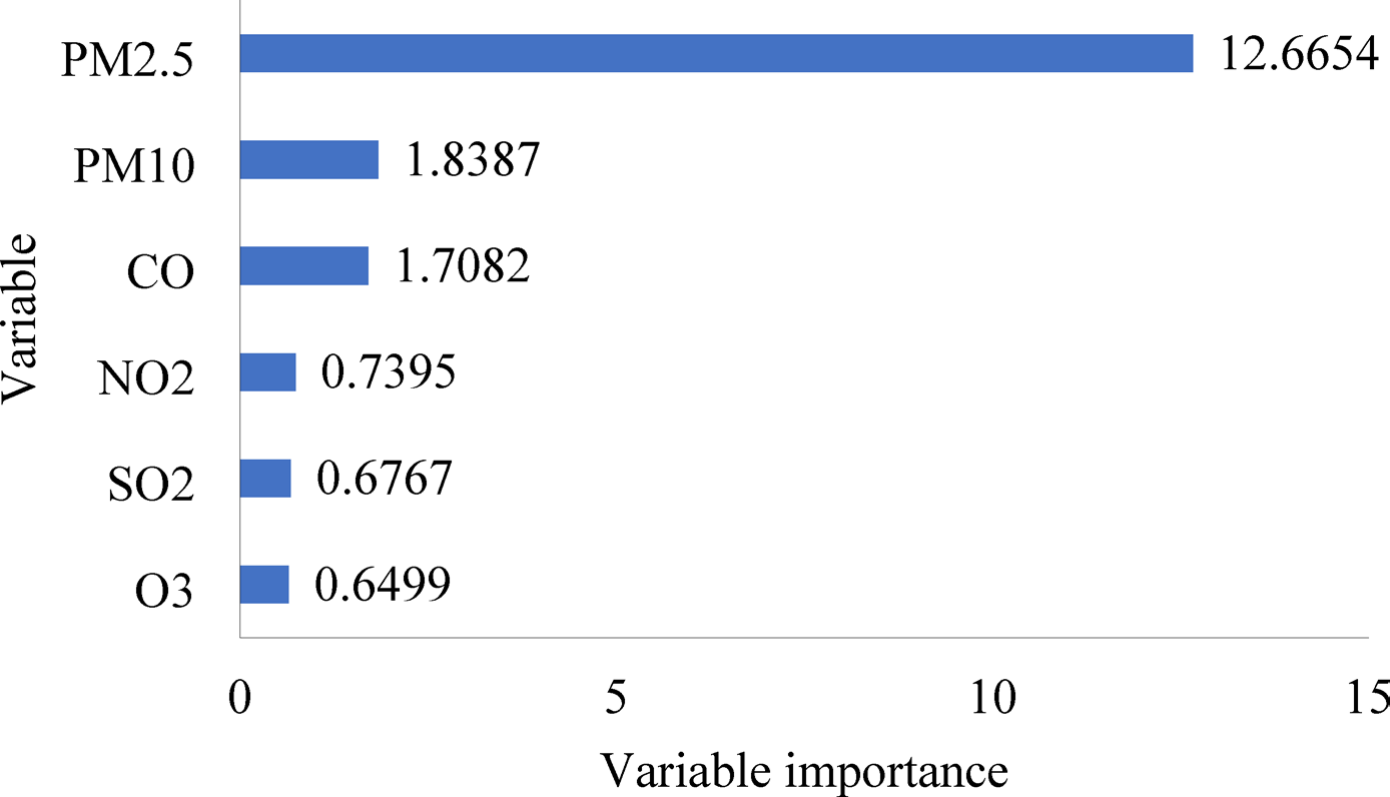
A bar chart for Random Forest variable importance.

To identify the most influential input variables for AQI prediction, a Random Forest was employed for feature importance evaluation. This technique effectively captures nonlinear relationships and interactions among variables, enabling a robust and data-driven approach to feature selection. The analysis revealed that PM_2.5_ had the highest importance score (12.6654), followed by PM_10_ (1.8387) and CO (1.7082). Although PM_2.5_ and PM_10_ exhibited a moderate correlation (r = 0.3014), computed using the Pearson correlation coefficient as defined in Eq. (2), both were retained due to their distinct and substantial contributions to AQI prediction. In contrast, NO_2_ (0.7395), SO_2_ (0.6767), and O_3_ (0.6499) demonstrated lower importance and were excluded from the final model. A bar chart summarizing these feature importance scores is presented in Fig. 3 to enhance clarity, transparency, and reproducibility of the variable selection process in alignment with best practices in machine learning-based environmental modeling.

2 $$r= \frac{\sum_{i=1}^{n}\left({x}_{i}- \overline{x }\right)({y}_{i}- \overline{y })}{\sqrt{\sum_{i=1}^{n}{({x}_{i}- \overline{x })}^{2}}. \sqrt{\sum_{i=1}^{n}{({y}_{i}- \overline{y })}^{2}}}$$

where:

$$r= \text{Pearson correlation coefficient}$$

$${x}_{i}= Individual values of pollutant x and y$$

$$\overline{x } and \overline{y }= Mean of x and y, respectively$$

n = Number of data points

### Developing and evaluating ML models

The Learner Regression App is a graphical interface provided within MATLAB’s Statistics and Machine Learning Toolbox^24^. Regression model development and analysis for use in predictive modeling tasks are made straightforward by this tool. The application provides an intuitive interface that facilitates interactive exploration and analysis of data, forecasting model construction, algorithm performance assessment, and prediction. This study utilizes regression techniques, including GPR, ER, SVM, RT, and KAR. Each model was selected based on its theoretical suitability and previous success in environmental prediction tasks. GPR provides probabilistic outputs and robustness to noise; ER enhances generalization by aggregating multiple base learners; SVM is well-suited for high-dimensional data spaces; RT offers interpretability and simplicity; and KAR strengthens the model’s ability to capture complex, nonlinear relationships.

Model training was conducted using standardized input variables (PM_2.5_, CO, and PM_10_), and hyperparameters were tuned to optimize performance. To ensure robustness and minimize overfitting, all models were cross-validated using tenfold cross-validation, a method that systematically partitions the data to reduce model bias and variance. Performance evaluation was carried out using established regression metrics, including R^2^, RMSE, and MAE. The detailed configurations and optimized hyperparameters applied for each model are summarized in Table 2.

**Table 2:** Applied Hyperparameters during the training phase.

Model	Parameters	Model	Parameters
GPR	Preset: Squared Exponential GPR	KAR	Preset: Least Squares Regression Kernel
	Basis function: Constant		Learner: Least Squares Kernel
	Kernel function: Squared Exponential		Number of expansion dimensions: 128
	Use isotropic kernel: Yes		Regularization strength (Lambda): 0.00023697
	Kernel scale: 0.12594		Kernel scale: 1
	Signal standard deviation: 0.063391		Standardize data: Yes
	Sigma: 0.063391		Iteration limit: 1000
	Standardize data: Yes		
	Optimize numeric parameters: Yes		
ER	Preset: Bagged Trees	RT	Preset: Coarse Tree
	Minimum leaf size: 8		Minimum leaf size: 36
	Number of learners: 30		Surrogate decision splits: Off
	Number of predictors to sample: 3		
SVM	Preset: Medium Gaussian SVM		
	Kernel function: Gaussian		
	Kernel function: 1.7		
	Box constraint: 0.074626		
	Epsilon: 0.0074626		
	Standardize data: Yes		

### Performance evaluation of machine learning models

The models’ evaluation is critical when utilizing ML to predict the AQI, so the learner regression tool provides three main metrics to assess it. These three metrics are Mean absolute error (MAE), Root mean square error (RMSE), and Determination coefficient (R^2^). The following equations can represent these statistical indicators:

- MAE.

This condition allows the error’s value to be measured in the forecast dataset while being heedless of directions. MAE reflects the average of absolute deviations between observed and predicted values across test samples. As can be calculated from Eq. (3):

3 $$MAE=\frac{1}{n}\sum_{i=1}^{n}\left|{x}_{i}-{y}_{i}\right|$$

where:

$n$ = Data points Number.

$${x}_{i}$$ = Actual value.

$${y}_{i}$$ = Predicted value.

(b) RMSE.

The RMSE is further used to estimate the value of the errors. To accomplish this, one also finds the square root of the latter by taking the mean of the square of the statistical variable in terms of the actual and predicted values as calculated in Eq. (4):

4 $$RMSE=\sqrt{\frac{1}{n}\sum_{i=1}^{n}{\left({x}_{i}-{y}_{i}\right)}^{2}}$$

where:

$${x}_{i}$$ = actual observation.

$${y}_{i}$$ = predicted values.

n = number of data points.

(c) R^2^.

The coefficient of determination represents a metric that assesses the extent to which a model accounts for the variance in observed data relative to its predictions. Specifically, it quantifies the proportion of total variability in actual values that the model’s predictions can explain. Its values range between 0 and 1, where a higher value suggests superior model performance. Conceptually, it is the ratio of variance explained by the model to the total variance observed in the data. An R-squared value nearing 1 indicates that the model’s predictions align with the actual data values. It can be calculated as shown in Eq. (5):

5 $${R}^{2}=1-\frac{{\sum }_{i=1}^{n}{\left({X}_{i}-{Y}_{i}\right)}^{2}}{\sum_{i=1}^{n}{\left({X}_{i}- \overline{X }\right)}^{2}}$$

where:

$${X}_{i}$$ = Actual values.

$${Y}_{i}$$ = Predicted values.

$\overline{X }$ = The mean of actual values.

*n* = Data points number.

## Results and discussion

### Air quality parameters

#### Gaseous pollutant

**Fig. 4:**
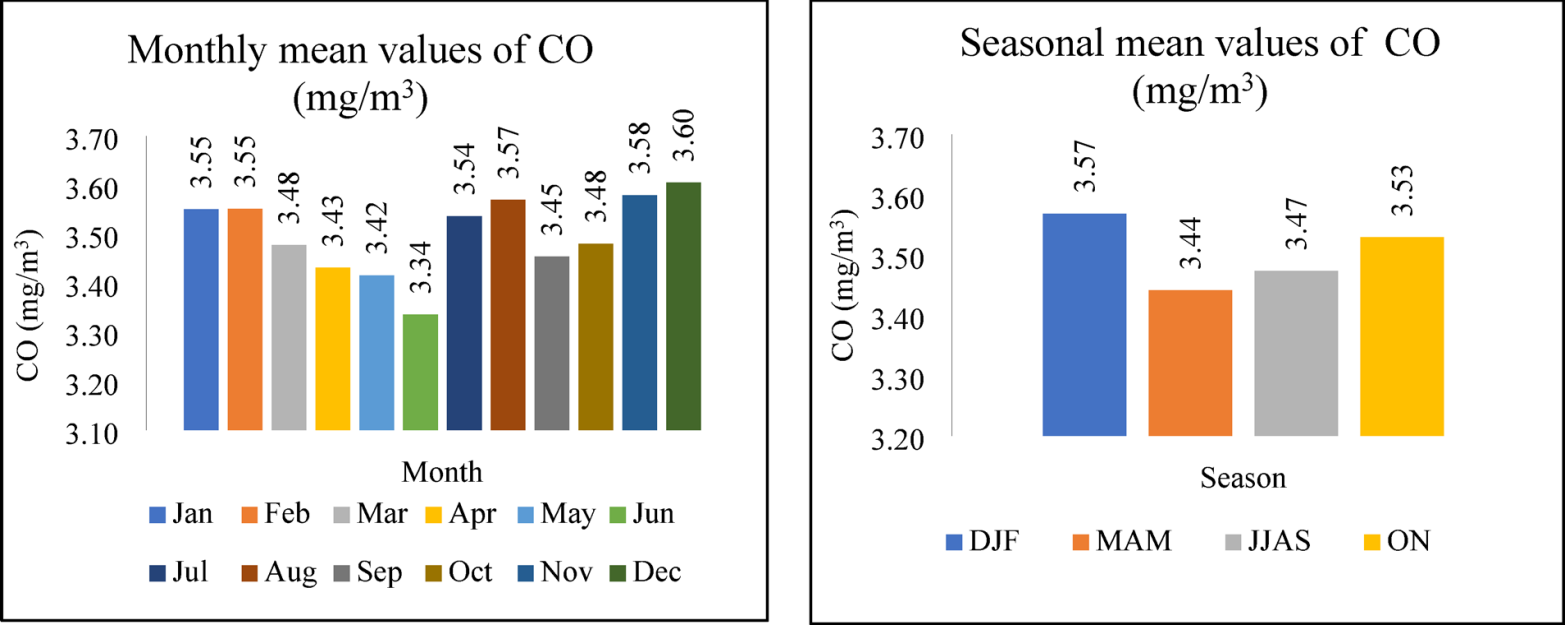
The variation of Monthly and Seasonal Gaseous pollutants (CO).

**Fig. 5:**
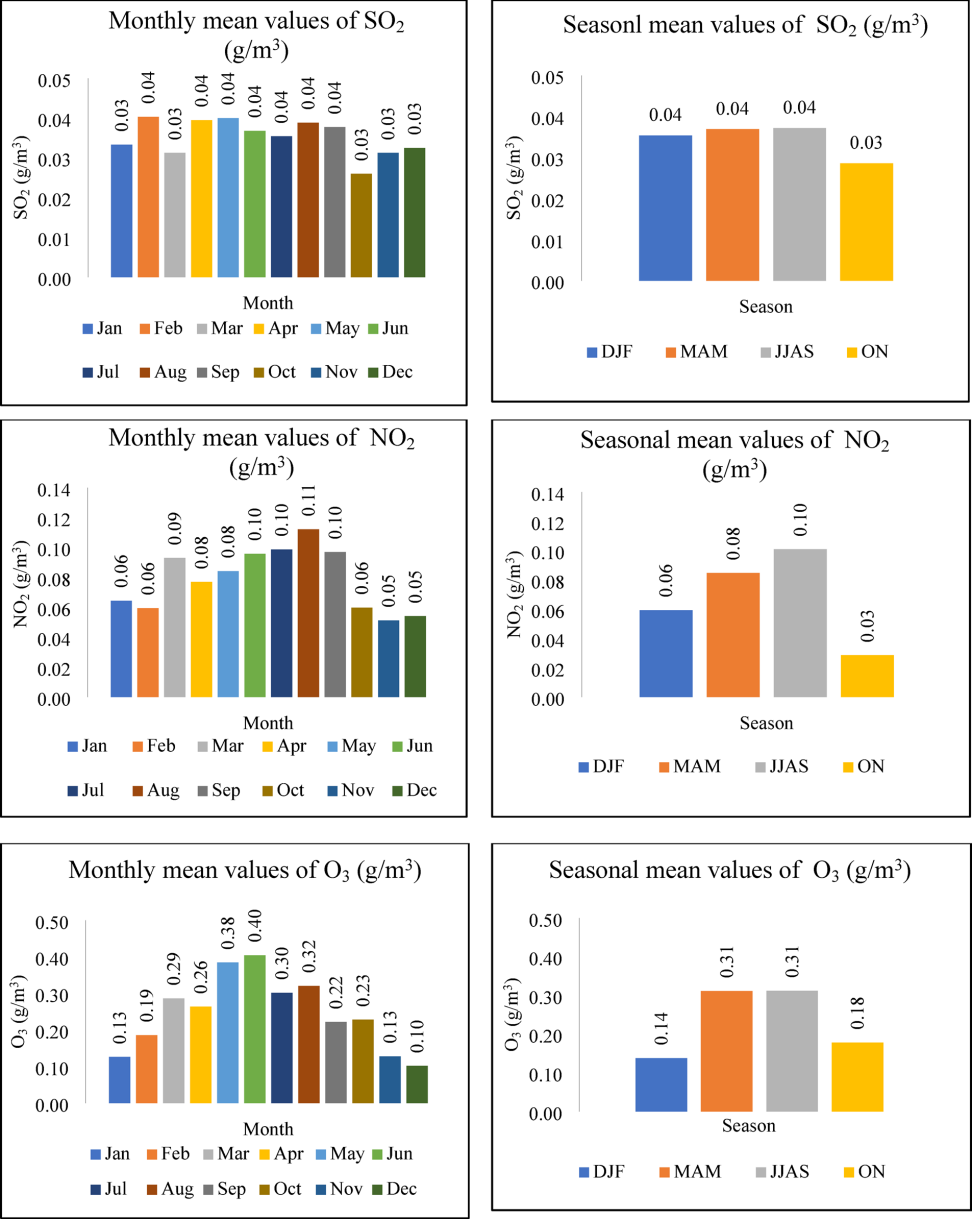
The variation of Monthly and Seasonal Gaseous pollutants (SO_2_, NO_2_, and O_3_).

Gaseous pollutants are a primary contributor to long-term atmospheric effects like climate change. When these gases are emitted and interact within the atmosphere, secondary air pollutants, in turn, are formed^25,26^. Figures 4 and 7 depict the monthly and seasonal variations of CO concentration in the atmosphere and AQI. For seasonal analysis, Winter (DJF: December–February), Summer (MAM: March–May), Monsoon (JJA: June–August), and Post-monsoon (SON: September–November) periods were considered, following the local climatological convention. It can be observed that both CO and AQI reach relatively higher values during the Winter and Post-Monsoon seasons, suggesting a possible seasonal association. However, this relationship is not consistent across all seasons and appears to be influenced by other contributing pollutants. In the case of SO_2_, NO_2_, and O_3_, the three pollutants follow the same pattern shown in Fig. 5. Their concentrations are higher in Summer and Monsoon than in Winter and Post-Monsoon; this phenomenon can be attributed to seasonal meteorological factors. High temperatures increased solar radiation, and higher humidity in these periods promote photochemical reactions that produce O_3_ and elevate NO_2_ and SO_2_ levels. During monsoon months, rainfall and wind patterns play a key role in the atmospheric distribution and removal of pollutants. SO_2_ concentrations tend to peak during the dry season and drop significantly in the wet season. This seasonal reduction is not only due to a decline in emissions from sources such as motor vehicles, brick production^27^, and industrial activity^28,29^, but also due to the washout effect of heavy monsoon rains, which efficiently scavenge gaseous pollutants like SO_2_ from the atmosphere. This wet deposition mechanism is especially effective in tropical regions such as Bangladesh, where intense rainfall is common during the monsoon period.

## Particulate matter (PM)

**Fig. 6:**
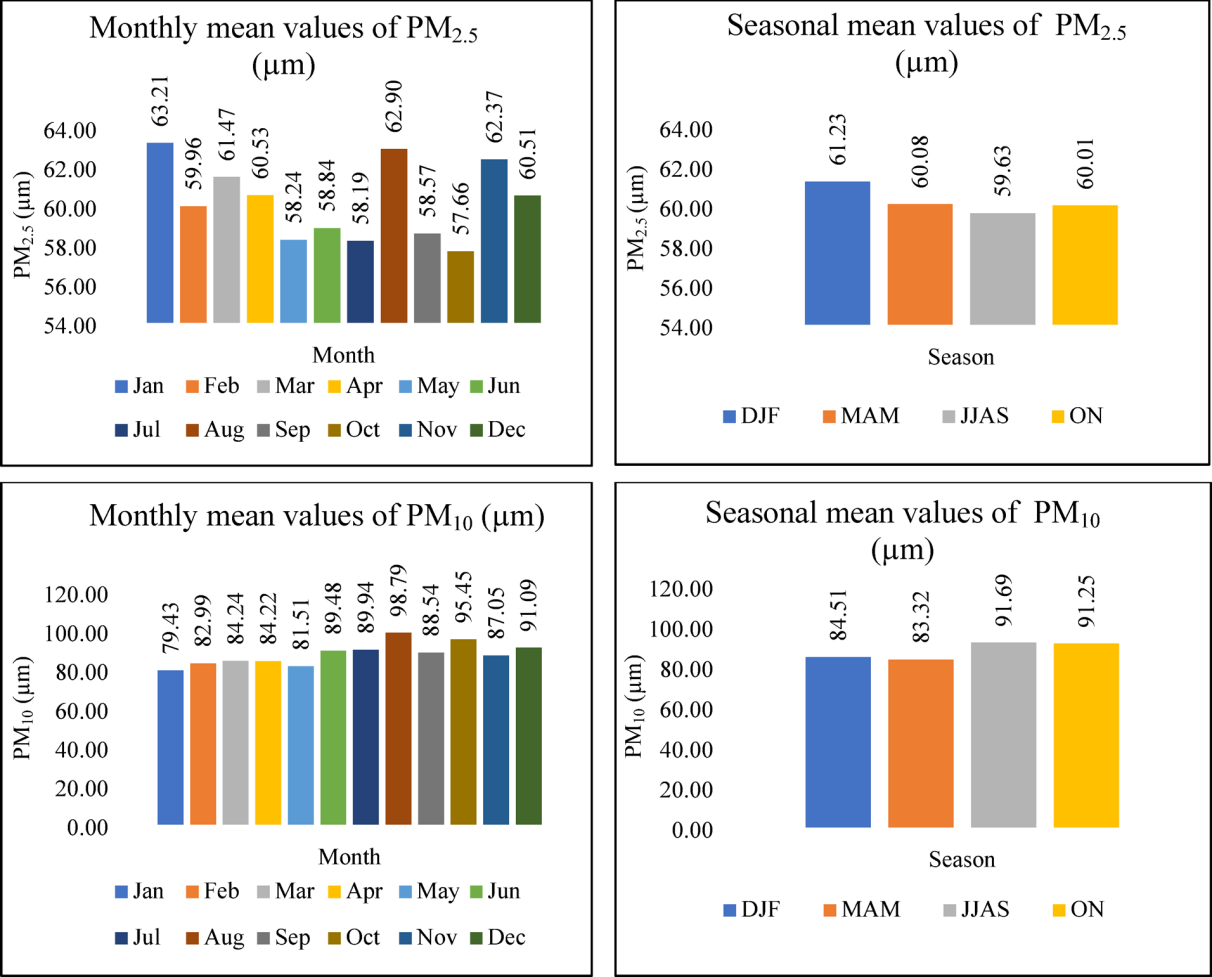
The variation of Monthly and Seasonal Particulate Matter (PM_2.5_ and PM_10_).

**Fig. 7:**
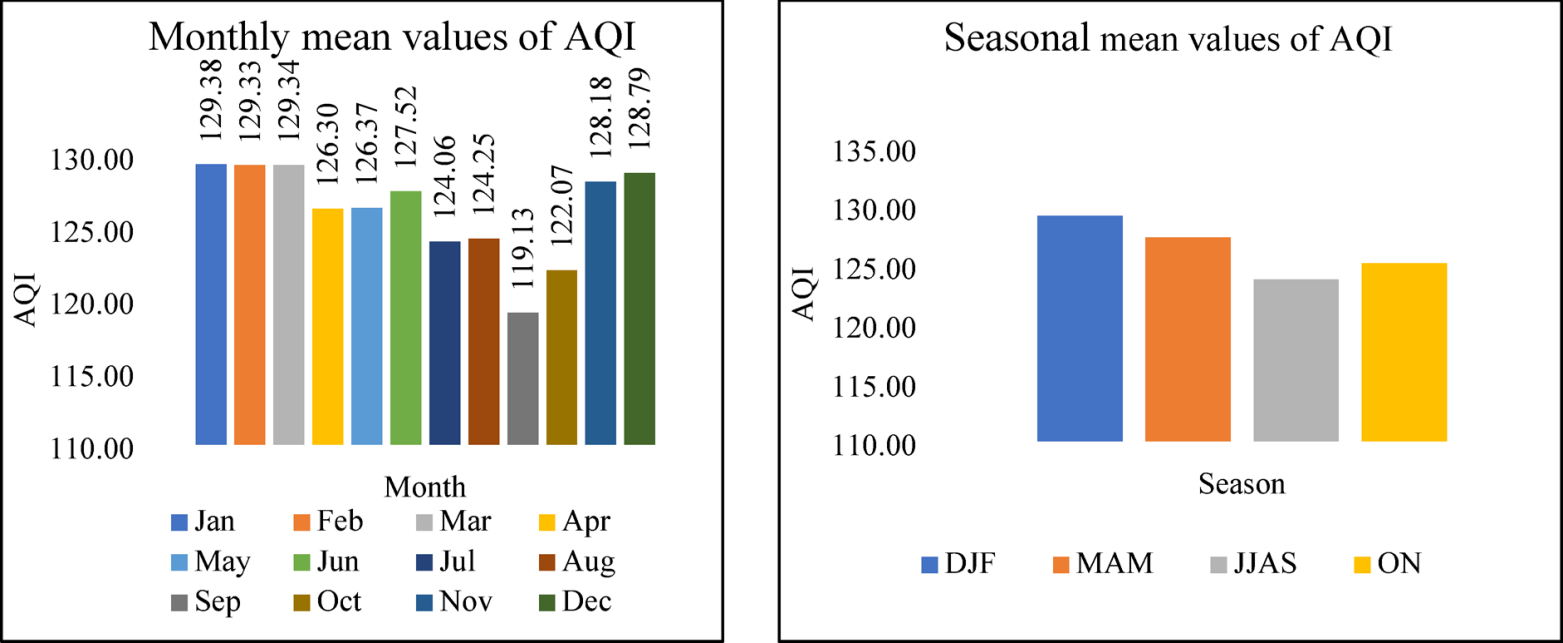
The variation of Monthly and Seasonal AQI.

Based on size, particulate matter can be divided into PM_10_ and PM_2.5_. These particles are primarily emitted from the construction, transportation, and thermal power plants worked by coal^30^. The respirable nature of particulate matter poses unavoidable risks to human well-being and plant life, allowing particles to penetrate the lungs and enter the bloodstream, which is the case for finer particles. Additionally, exposure to these fine particles is linked to an increased risk of cancer. Consequently, particulate matter is essential for assessing the AQI^31^. Figure 6 illustrates the monthly and seasonal fluctuations of PM_2.5_ and PM_10_ levels. Analysis of Figs. 6 and 7 reveals a similar trend between the AQI and PM_2.5_ concentrations, suggesting a significant influence of PM_2.5_ on AQI calculations. The PM_2.5_ trend mirrors the AQI’s, with peak concentrations occurring in the Winter months. This seasonal increase is attributed to the accumulation of PM_2.5_ in the atmosphere during Winter, leading to elevated atmospheric concentrations^32^. On the other hand, it is apparent from Figs. 6 and 7 that PM_10_ and AQI follow the same pattern in post-monsoon, influencing the AQI.

## AQI

Differences in monthly AQI are shown on Fig. 7. The results depict that the AQI value was higher than 100, and its range is between 119 and 130, indicating that Gazipur City is moderately polluted. December to February, which falls under the Winter period, shows slightly high AQI values, reaching 129.168. The AQI levels are high in these months due to seasonal variation^33^. During the winter, cooler temperatures and increased mist formation contribute to atmospheric conditions conducive to temperature inversions. These inversions, sustained by moist air, prevent the dispersal of pollutants, causing them to accumulate in the atmosphere. With denser air during winter, the limited atmospheric space restricts the escape of pollutants, leading to an elevated concentration of airborne contaminants^34^. Furthermore, the range of the AQI improved from July to September to 119.134, as shown in the results. In Bangladesh, the months from June to September typically experience significant rainfall, driven by the southwest monsoon winds, with peak precipitation occurring at high intensities^35^. Due to rains, PM_2.5_ and other gaseous pollutants settle; as a result, lower air pollutant concentrations in the atmosphere occur.

### Machine learning models for predicting AQI

ML models were developed using the MATLAB learner regression tool after data preparation, cleaning, normalization, and parameter selection. The acceptable 7550 samples data have been divided by 80% (6040 samples) for training and 20% (1510) for testing. The model performance evaluation was conducted during the training and testing phases. The results are presented in Table 3.

**Table 3:** Evaluation of model performance during both training and testing phases.

Parameter	Statistical index	Training	Testing
GPR	R^2^	0.9957	0.9913
	RMSE	0.8781	1.2179
	MAE	0.1785	0.2722
ER	R^2^	0.9950	0.9908
	RMSE	0.9495	1.2512
	MAE	0.0960	0.1540
SVM	R^2^	0.9945	0.9937
	RMSE	0.9944	1.0372
	MAE	0.7491	0.7760
RT	R^2^	0.9744	0.9643
	RMSE	2.1370	2.4697
	MAE	0.3892	0.4709
KAR	R^2^	0.9744	0.8236
	RMSE	2.1374	5.4928
	MAE	1.8540	2.6311

### Comparative analysis of AQI prediction models

To evaluate the predictive effectiveness of the five regression models—GPR, ER, SVM, RT, and KAR—a comparative analysis was performed using standard metrics: R^2^, RMSE, and MAE for both training and testing phases. Additionally, the analysis considered each model’s generalization ability, individual prediction errors, and structural behavior under unseen conditions.

In the training phase, GPR exhibited the strongest performance (R^2^ = 0.9957, RMSE = 0.8781, MAE = 0.1785), suggesting highly accurate modeling and low deviation from actual AQI values. ER followed with similarly robust performance (R^2^ = 0.9950, RMSE = 0.9495, MAE = 0.0960), indicating excellent variance reduction due to bagging multiple learners. SVM achieved a high R^2^ (0.9945) but had a relatively higher MAE (0.7491), highlighting increased individual prediction errors despite capturing overall trends effectively. RT (R^2^ = 0.9744, RMSE = 2.1370, MAE = 0.3892) and KAR (R^2^ = 0.9744, RMSE = 2.1374, MAE = 1.8540) showed moderate accuracy in training but higher MAE values, suggesting overfitting and poor error control on a granular level.

**Fig. 8:**
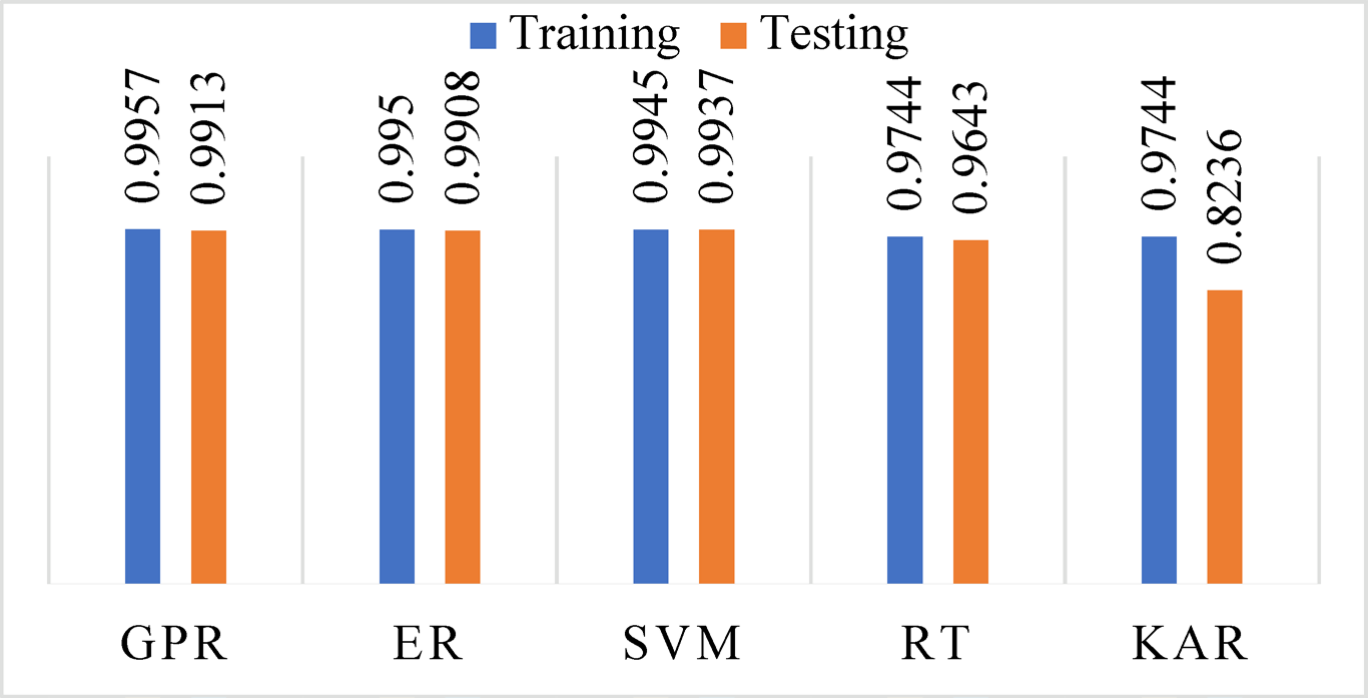
R^2^ during training and testing stages.

**Fig. 9:**
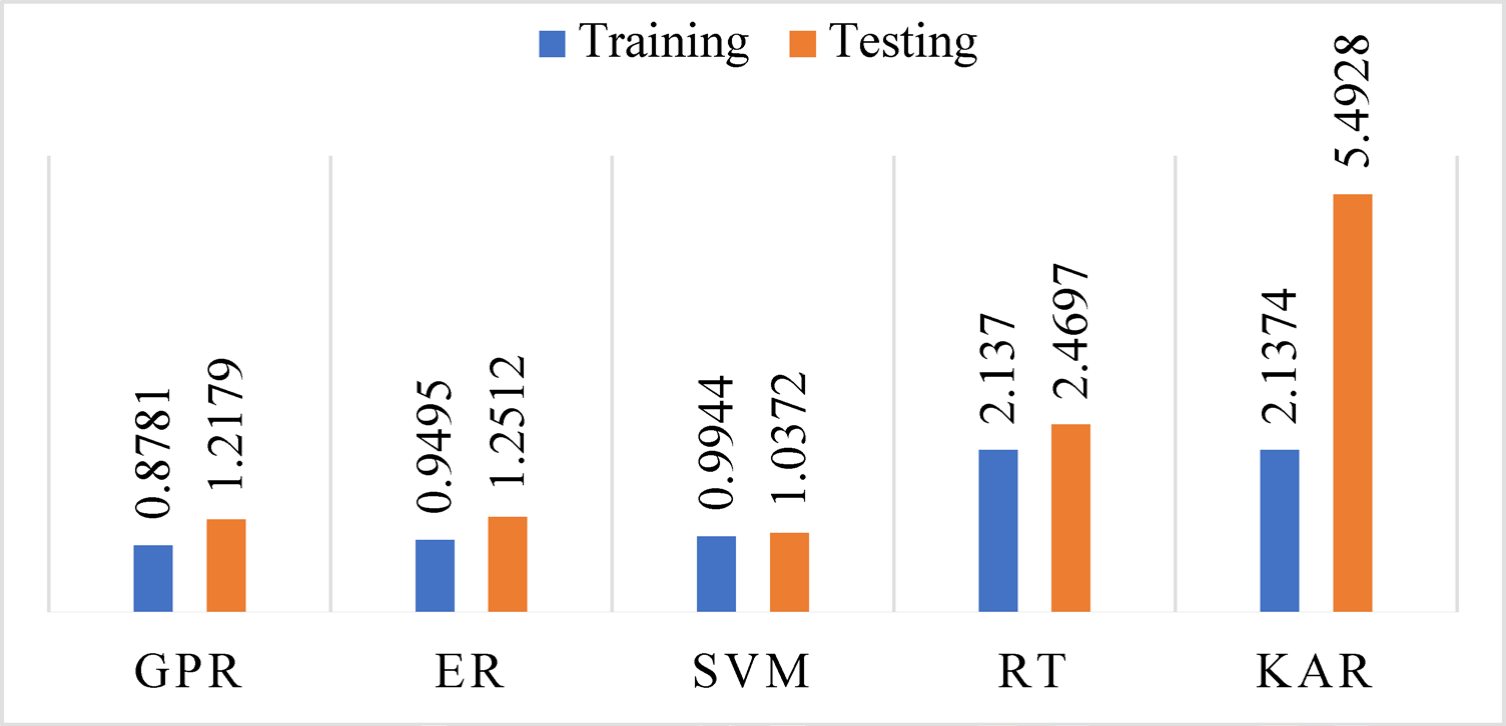
RMSE during training and testing stages.

**Fig. 10:**
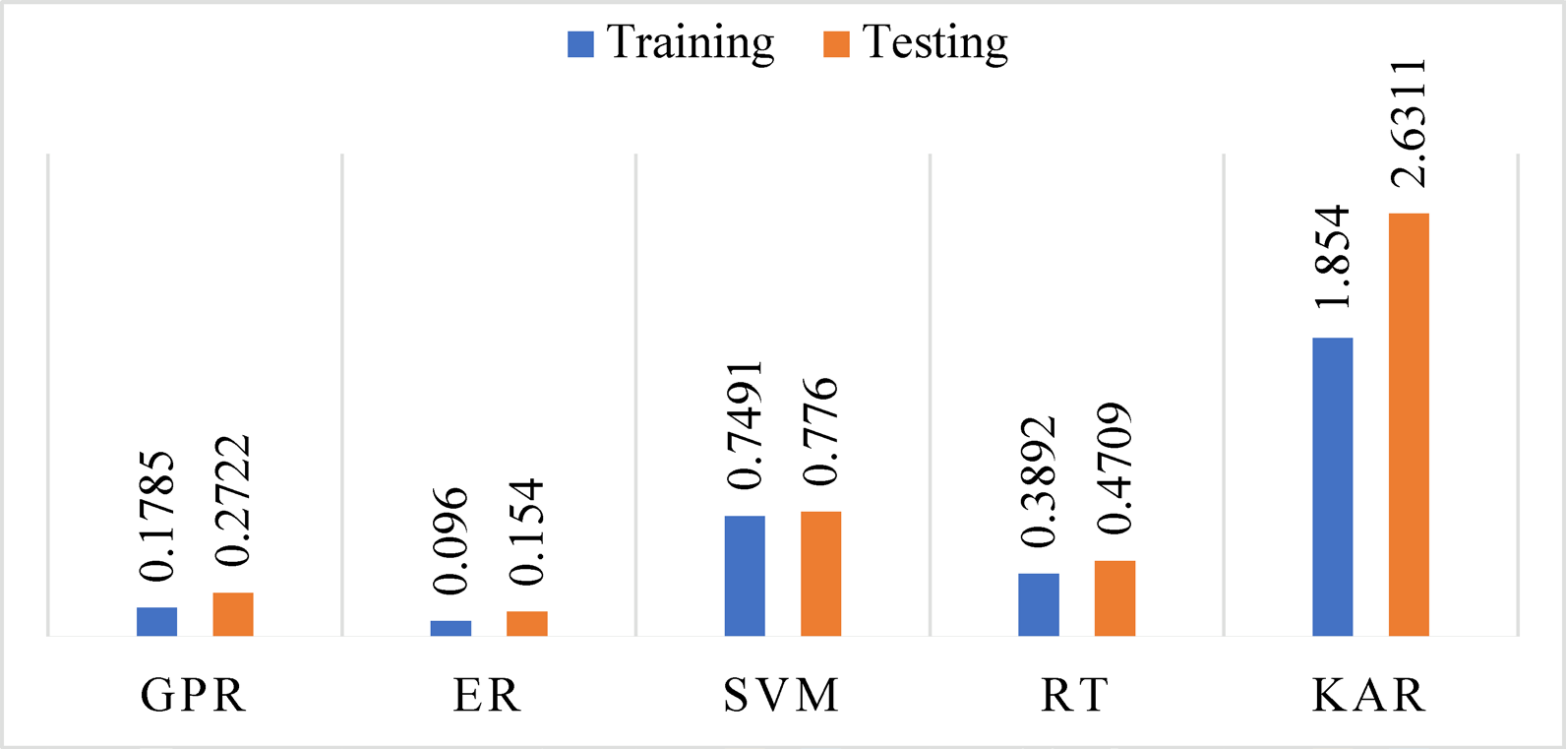
MAE during training and testing stages.

**Fig. 11:**
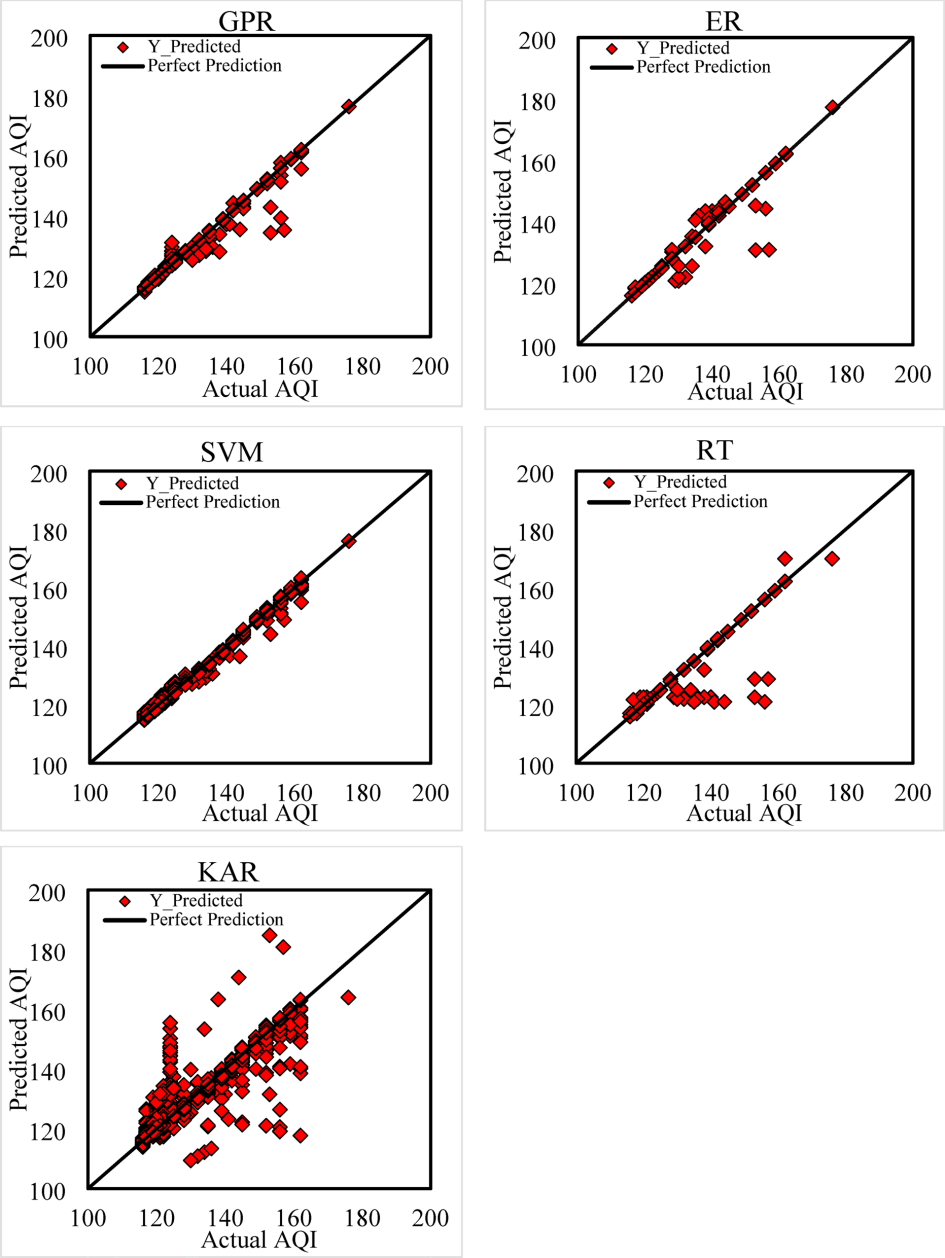
Comparison between observed and predicted AQI values (testing phase) for GPR, ER, SVM, RT, and KAR.

In the testing phase (see Figs. 8, 9, 10 and 11), SVM surprisingly outperformed all other models with an R^2^ of 0.9937 and RMSE of 1.0372, though its MAE remained high (0.7760), implying uneven performance on specific predictions. GPR and ER retained strong predictive power (GPR: R^2^ = 0.9913, RMSE = 1.2179, MAE = 0.2722; ER: R^2^ = 0.9908, RMSE = 1.2512, MAE = 0.1540), demonstrating strong generalization with low error margins. In contrast, RT (R^2^ = 0.9643, RMSE = 2.4697, MAE = 0.4709) experienced a notable decline in accuracy due to structural overfitting. KAR suffered the most significant deterioration (R^2^ = 0.8236, RMSE = 5.4928, MAE = 2.6311), indicating its reduced adaptability to new data and limitations in capturing non-linearities when trained on limited feature dimensions (Fig. 11).

To explore model robustness, a tenfold cross-validation technique was applied, and 80% of the data was used for training while 20% was reserved for testing. The high performance of GPR and ER across both phases confirms their stability and minimal overfitting. In contrast, the severe decline in KAR’s testing performance suggests structural limitations, possibly due to sensitivity to noise, poor regularization in high-dimensional mapping, or lack of sufficient training support for kernel expansion dimensions.

## Conclusion

The predicted AQI was examined for Gazipur based on historical data collected from 1st January 2022 to 31st December 2022. The highest AQI value (129) occurred during Winter due to low temperatures and mist formation that trap pollutants near the ground, while the lowest value (123) appeared during the Monsoon season because of rainfall-induced pollutant dispersion. Among the input features, PM_2.5_ had the highest feature importance score (12.6654), confirming its dominant influence on AQI in urban areas. PM_10_ (1.8387) and CO (1.7082) also played meaningful roles, while NO_2_, SO_2_, and O_3_ showed minimal impact with lower scores. Therefore, to balance model performance and practicality, the final AQI prediction model was developed using only PM_2.5_, PM_10_, and CO. This choice supports a more efficient, scalable, and cost-effective monitoring system that is well-suited for real-world deployment, especially in developing urban settings where comprehensive pollutant tracking may not be feasible. ML models effectively predicted the AQI: GPR, ER, SVM, RT, and KAR, showing maximum correlations of 0.9957, 0.9950, 0.9945, 0.9744, and 0.9744, respectively, for training datasets, and 0.9913, 0.9908, 0.9937, 0.9643, and 0.8236, for testing datasets. Therefore, this analytical study is valuable for assessing the effectiveness of selected models in specific contexts. Moreover, it provides insights into the effectiveness of various predictive models for air quality, supporting policymakers and urban planners in creating strategies to address air pollution.

### Limitations and future work

This study is limited to a one-year dataset from a single city, which may restrict the generalizability of the findings across different regions or time frames. The exclusion of meteorological variables such as wind speed and humidity may also affect the model’s accuracy under varying environmental conditions. Although PM_2.5_, PM_10_, and CO were prioritized based on their importance and practicality, other pollutants such as SO_2_, NO_2_, and O_3_ may hold greater significance in different geographic or seasonal contexts. Their exclusion was intended to optimize efficiency, not to diminish their potential relevance.

Future research should apply formal statistical tests, such as ANOVA or t-tests, to assess the significance of performance differences across models.

## Data Availability

The datasets used and/or analysed during the current study are available from the corresponding author upon reasonable request.
